# Biological Augmentation of Reamed Intramedullary Nailing for Aseptic Tibial Shaft Nonunion: An Exploratory Multicenter Retrospective Comparative Cohort Study

**DOI:** 10.3390/jfmk11020239

**Published:** 2026-06-16

**Authors:** Michele Coviello, Luigi Meccariello, Giuseppe Rovere, Vincenzo Caiaffa, Giuseppe Rollo, Francesco Liuzza, Mario Ronga, Francesco Ippolito, Amarildo Smakaj, Maria Lucia Mancini, Antonio Colella, Giuseppe Maccagnano

**Affiliations:** 1 Orthopaedics Unit, Department of Clinical and Experimental Medicine, Faculty of Medicine and Surgery, University of Foggia, Policlinico Riuniti di Foggia, 71122 Foggia, Italy; giuseppe.maccagnano@unifg.it; 2 Department of Orthopedics and Traumatology, AORN San Pio Hospital, 82100 Benevento, Italy; 3 School of Medicine and Surgery, University of Doboj, 74000 Doboj, Bosnia and Herzegovina; 4 Department of Clinical Science and Translational Medicine, Section of Orthopaedics and Traumatology, University of Rome Tor Vergata, 00133 Rome, Italy; 5 Department of Orthopaedics and Traumatology, Di Venere Hospital, 70131 Bari, Italy; 6 Department of Orthopedics and Traumatology, Vito Fazzi Hospital, 73100 Lecce, Italy; 7 Orthopaedic and Trauma Operative Unit, Department of Health Sciences, University of Eastern Piedmont, 28100 Novara, Italy; 8 Department of Orthopedics and Traumatology, San Giuseppe Da Copertino Hospital, 73043 Copertino, Italy; 9 Department of Pediatric Orthopedics, Children’s Hospital Giovanni XXIII, 70126 Bari, Italy; antonio.colella@gmail.com

**Keywords:** nonunion, teriparatide, fat, adipose derived stem cells, ADSCs, outcomes, ASAMI, nail, limb reconstruction, stem cell

## Abstract

**Background**: Despite stable fixation, aseptic tibial shaft nonunion represents a severe orthopedic complication. Teriparatide and adipose-derived stem-cell augmentation have been proposed as biological supports, but comparative clinical evidence remains limited. This study explored whether adding adjuvant therapies to reamed intramedullary nailing was associated with faster healing than nailing alone. **Methods:** We retrospectively reviewed 43 adults with aseptic tibial shaft nonunion treated at three level I trauma centers between 2017 and 2020. Patients underwent reamed intramedullary nailing alone (*n* = 15), nailing plus teriparatide (*n* = 15), or nailing plus adipose-derived stem-cell augmentation (*n* = 13). Group allocation was nonrandom and based on contraindications and patient acceptance; results were therefore interpreted as exploratory. Outcomes included time to tricortical radiographic healing, pain, radiographic healing scores over time, complications, ASAMI classification, and SF-12. **Results:** Baseline demographic and fracture characteristics were comparable across groups. Time to tricortical radiographic healing was shorter in the teriparatide group (87.46 ± 6.34 days) and the adipose-derived stem-cell group (86.41 ± 5.67 days) than in the nailing-alone group (99.71 ± 4.29 days; *p* = 0.034). Pain, complication rates, ASAMI outcomes, and quality-of-life recovery did not differ significantly among groups at final follow-up. **Conclusions:** In this exploratory multicenter retrospective cohort, teriparatide and adipose-derived stem-cell augmentation were associated with shorter radiographic healing time after reamed intramedullary nailing for aseptic tibial shaft nonunion, but not with superior long-term functional outcomes. Because no comparator group treated with standard graft-based biological augmentation was included, the findings should be interpreted cautiously. Larger prospective studies or randomized controlled trials comparing these strategies with established graft-based approaches are needed to validate the present data.

## 1. Introduction

Aseptic tibial shaft nonunion remains one of the most challenging problems in orthopedic trauma surgery. Although most tibial shaft fractures are successfully treated, a delayed union or nonunion, especially after high-energy trauma, severe soft-tissue injury, or repeated surgical procedures could occur [1,2,3,4,5]. Above its biological and mechanical complexity, tibial nonunion poses a major challenge to patients and healthcare systems since it is associated with persistent pain, prolonged disability, recurrent surgeries, delayed return to work, and increased treatment costs [3,4].

The management of aseptic tibial shaft nonunion is based on correction of the factors that retard bone healing. These commonly include insufficient mechanical stability, inadequate biological activity at the nonunion site, altered vascularity, bone loss, and unfavorable host-related factors [6,7,8,9]. In current clinical practice, reamed intramedullary exchange nailing is a well-established strategy for aseptic tibial shaft nonunion because it can restore axial and rotational stability while also providing biological stimulation through medullary reaming [10,11,12]. However, even when fixation is adequate, some nonunions may remain biologically compromised and may therefore benefit from additional biological stimulation.

This concept is consistent with the “diamond” model of fracture healing, according to which successful consolidation depends on the interaction among mechanical stability, osteogenic cells, osteoconductive support, osteoinductive mediators, and vascularity [8,9]. Within this setting, biological augmentation may be particularly useful in selected aseptic nonunions in which stable fixation alone may not adequately address the compromised regeneration environment. Consequently, the integration of a standardized mechanical treatment with an adjuvant biological strategy may represent a rational approach in carefully selected individuals.

Although the risk of nonunion in tibial fractures is widely acknowledged, treatment with reamed intramedullary nailing for closed fractures has shown relatively low nonunion rates, reported between 0% and 4%. In cases requiring large bone resections, Ilizarov’s osteo-distraction technique is often employed as part of the treatment approach [11]. Additionally, intramedullary nailing has become a common method for treating aseptic nonunions. In standard orthopedic trauma practice, the management of aseptic nonunion of the tibia is fairly well established and involves replacement of the nail in cases of hypertrophic pseudarthrosis and the use of a biological stimulus such as bone grafting (often autologous from the iliac crest or other femoral substitutes) in atrophic cases [13,14].

Among the biological agents proposed for this purpose, teriparatide has attracted growing interest. Teriparatide is a recombinant human parathyroid hormone fragment (PTH) with anabolic effects on bone metabolism. Experimental and clinical studies suggest that intermittent administration may stimulate osteoblast activity, enhance callus formation, and promote endochondral ossification, thereby potentially supporting fracture repair and nonunion healing [15,16,17,18,19]. Nevertheless, the currently available clinical evidence is still limited, heterogeneous, and largely based on case reports, case series, or studies performed in fracture settings other than tibial shaft nonunion [15,18,19].

Adipose-derived stem cells (ADSCs) have also been investigated as a possible biological adjunct in bone regeneration. Adipose tissue provides an accessible source of mesenchymal stromal cells with osteogenic and angiogenic potential, and stromal vascular fraction preparations may contribute to healing through both progenitor-cell activity and paracrine signaling [20,21,22]. However, clinical evidence supporting the use of ADSCs in aseptic long-bone nonunion remains preliminary, with small and heterogeneous studies that do not yet allow firm conclusions regarding indications, standardization, or magnitude of benefit [21,23].

For these reasons, the role of biological augmentation in aseptic tibial shaft nonunion has not yet been clearly defined. It is unclear, in particular, whether adding teriparatide or ADSCs to reamed intramedullary nailing gives clinically substantial improvements over nailing alone, other than possibly accelerating radiographic recovery. The present multicenter retrospective comparative cohort study was therefore designed to explore the outcomes of three treatment strategies for aseptic tibial shaft nonunion: reamed intramedullary nailing alone, nailing combined with teriparatide, and nailing combined with ADSC augmentation. We hypothesized that biological augmentation might be associated with faster radiographic consolidation, while long-term functional outcomes could remain comparable among groups.

## 2. Materials and Methods

### 2.1. Study Design and Ethical Approval

This was a multicenter retrospective comparative cohort study including patients surgically treated for aseptic tibial shaft nonunion between January 2017 and December 2020 at three orthopedic Level I trauma centers. The study was conducted in accordance with the Declaration of Helsinki. Ethical approval (protocol code 0326, approved on 20 February 2026) was obtained before retrospective review, extraction, and analysis of clinical data for research purposes. All patients had been treated as part of routine clinical practice, and no diagnostic or therapeutic procedures were performed specifically for this study. Clinical information was retrospectively collected from institutional medical records and anonymized before analysis in accordance with applicable ethical and privacy regulations.

### 2.2. Patient Selection

Patients were eligible if they had an aseptic tibial shaft nonunion following a fracture classified as AO/OTA 42 [24] and were treated with reamed intramedullary nailing as part of the revision procedure with a minimum clinical and radiographic follow-up of 12 months after nonunion surgery. Nonunions were classified according to the ASAMI system, and only type B nonunions were included. To describe baseline severity, the Nonunion Scoring System (NUSS) was retrospectively calculated for each case [25].

Exclusion criteria were active or previous infection at the nonunion site, osteoporotic or metabolic bone disease, hematological or oncological disease, previous major trauma of the same lower limb, associated nerve or vascular injury, skeletal immaturity, age younger than 16 years, amputated or threatened lower limb, definitive treatment with external fixation, and severe neurological or psychiatric disorders interfering with follow-up or rehabilitation.

The diagnosis of aseptic nonunion was established through a combination of clinical, laboratory, radiographic, and intraoperative findings. Clinically, patients had no local signs of infection such as erythema, drainage, sinus tract formation, or persistent wound complications. Laboratory evaluation included white blood cell count, C-reactive protein (CRP), and erythrocyte sedimentation rate (ESR), which were within normal limits or showed no pattern suggestive of active infection.

Given the high proportion of patients with a history of open fractures, particular attention was paid to excluding occult infection. During revision surgery, multiple deep tissue samples were routinely collected before antibiotic administration and submitted for microbiological culture according to institutional protocols. Patients with positive microbiological findings or clinical evidence suggestive of fracture-related infection were excluded from the study.

Despite this standardized assessment, the possibility of occult low-grade infection cannot be completely excluded, as is the case in all retrospective nonunion studies.

Standard radiographic assessment was performed in all cases, and advanced imaging was obtained when clinically indicated. Patients with positive cultures or findings suggestive of fracture-related infection were excluded from the study [25].

A total of 43 patients met the eligibility criteria and were included in the analysis. Of these, 15 were treated with reamed intramedullary nailing alone (Control group), 15 with reamed intramedullary nailing plus teriparatide (Teri group), and 13 with reamed intramedullary nailing plus adipose-derived stem-cell augmentation (ADSC group).

### 2.3. Treatment Allocation

Treatment allocation was not randomized. The choice of adjunctive biological treatment was based on clinical eligibility, contraindications, technical feasibility, and patient preference after multidisciplinary discussion with the treating surgeons.

Patients assigned to the teriparatide group were considered suitable for off-label anabolic therapy in the absence of contraindications such as previous skeletal malignancy, metabolic bone disorders, hypercalcemia, severe renal impairment, previous radiation therapy involving the skeleton, or known hypersensitivity to teriparatide. In addition, some patients declined teriparatide because of the need for daily subcutaneous injections or concerns regarding prolonged pharmacological treatment.

Patients assigned to the ADSC group were selected when adipose tissue harvesting was considered technically feasible and acceptable to the patient. Factors influencing refusal or non-feasibility of ADSC harvesting included limited abdominal adipose tissue, concerns regarding the additional harvesting procedure, reluctance toward liposuction-related morbidity, or preference to avoid additional surgical manipulation.

Patients in the control group generally included individuals who either declined adjunctive biological procedures, had relative contraindications to teriparatide, were not suitable candidates for adipose harvesting, or underwent standard revision nailing according to surgeon and patient preference.

Because treatment allocation depended partly on patient preference and clinical judgment, group comparisons were considered exploratory and interpreted with awareness of potential selection bias and residual confounding.

### 2.4. Surgical Technique and Postoperative Rehabilitation Protocol

In all patients, the surgical strategy aimed to restore mechanical stability and optimize the biological environment of the nonunion site. Fracture stability was assessed preoperatively. Nonunion was considered mechanically stiff when angular bending was less than 7° and/or axial displacement was less than 5 mm, according to established biomechanical criteria [6].

After surgical exposure, the nonunion site was debrided, either through an open or minimally open approach depending on the case, until viable bleeding bone was identified according to the “paprika sign” principle [7]. The medullary canal was then reamed to improve both mechanical fixation and biological stimulation [7,12]. A locked intramedullary nail was inserted in all cases, and compression across the nonunion site was applied whenever feasible in order to enhance stability and promote callus formation [26].

A fibular osteotomy was systematically performed before tibial reduction and compression, with the aim of reducing strain shielding and facilitating compression at the tibial nonunion site [27,28]. Nail fixation was completed using proximal and distal locking screws to provide axial and rotational stability.

All patients received perioperative antibiotic prophylaxis with cefazolin 1 g twice daily for 3 days, according to local orthopedic practice and guidelines [29]. Passive and active mobilization of the knee and ankle was started on the second postoperative day [30]. Full weight-bearing was encouraged as tolerated, unless specific clinical conditions required a more cautious progression [31].

### 2.5. Teriparatide Protocol

Patients assigned to the Teri group received teriparatide at a dose of 20 μg once daily by subcutaneous injection for 3 months after surgery [15,16,17,18,19,32]. This regimen was selected because it corresponds to the approved osteoporosis dosage and is the most commonly reported protocol in the literature investigating fracture healing, delayed union, and nonunion.

Teriparatide was prescribed off-label for nonunion treatment, and all patients received specific counseling before treatment initiation. Existing antiresorptive therapy, when present, was discontinued before teriparatide administration. Vitamin D deficiency was corrected, and calcium intake was optimized when necessary, according to current recommendations [33].

Treatment adherence was assessed during scheduled outpatient visits through direct patient interview and prescription review. All patients completed the planned 3-month treatment course. No treatment discontinuations or serious adverse events related to teriparatide administration were recorded during the study period.

### 2.6. ADSC Harvesting and Processing

In the ADSC group, autologous adipose tissue was harvested intraoperatively from the abdominal region by standard lipoaspiration. The 40–60 mL of lipoaspirate was processed in a sterile operating-room environment using ultrasonic cavitation and centrifugation to obtain a minimally manipulated stromal vascular fraction enriched with adipose-derived regenerative cells according to the institutional protocol shared among participating centers in order to obtain a freshly isolated stromal vascular fraction enriched with adipose-derived regenerative cells. The final preparation was not culture-expanded and was implanted during the same surgical procedure. Cell viability assessment was performed as per protocol, which demonstrated high viability of the final product before implantation. However, quantitative cell counts and detailed immunophenotypic characterization by flow cytometry (e.g., CD73, CD90, CD105, CD34, CD45 markers) were not systematically performed in all cases because of the retrospective multicenter design and the clinical-use setting. Therefore, the exact cellular composition and degree of progenitor-cell enrichment could not be standardized across centers. This limitation should be considered when interpreting reproducibility and biological comparability of the ADSC preparation, particularly because stromal vascular fraction products may vary depending on harvesting technique, processing protocol, and operator-dependent factors. According to the original protocol, the final cell product showed high viability and was applied locally as biological augmentation at the nonunion site [21].

### 2.7. Outcomes and Follow-Up

The primary outcome was time to tricortical radiographic healing, expressed in days from nonunion surgery to the first radiographic evidence of healing in at least three cortices.

Secondary outcomes included radiographic progression of healing, pain, perioperative and postoperative complications, functional outcome, and health-related quality of life.

Standardized anteroposterior and lateral radiographs of the tibia were obtained preoperatively, immediately after surgery, and at 1, 3, 6, and 12 months of follow-up. Tricortical radiographic healing was defined as the presence of bridging callus and cortical continuity in at least three of the four cortices visible on the anteroposterior and lateral projections.

Radiographic healing was independently assessed by two experienced orthopedic surgeons who were not involved in the index procedure. Healing progression was evaluated using the Radiographic Union Score for Tibial fractures (RUST) [19]. Interobserver agreement for RUST assessment was excellent (κ = 0.87).

In cases of disagreement, images were reviewed jointly and a consensus assessment was reached. When plain radiographs were considered equivocal or when confirmation of tricortical healing was uncertain, computed tomography was performed as an adjunctive imaging modality to confirm cortical bridging and bone consolidation.

Pain was assessed at each follow-up visit using the visual analog scale (VAS) [34]. Operative time, intraoperative blood loss, and complications were also recorded. At final follow-up, bone and functional outcomes were classified according to the ASAMI criteria [35,36], and patient-reported quality of life was assessed using the 12-Item Short Form Health Survey (SF-12).

### 2.8. Statistical Analysis

Descriptive statistics were used to summarize baseline characteristics, operative variables, and follow-up outcomes. Continuous variables were reported as mean and standard deviation, whereas categorical variables were reported as absolute numbers and percentages.

Because of the limited sample size and the comparison among three groups, nonparametric methods were preferred for most between-group analyses. Continuous variables were compared using the Kruskal–Wallis test, with post hoc pairwise comparisons performed when appropriate. Categorical variables were compared using the chi-square test or Fisher’s exact test when expected frequencies were small. Changes in radiographic and clinical scores over time were analyzed within groups using repeated-measures nonparametric testing as appropriate.

The association between radiographic healing and pain was explored by correlation and regression analysis. In view of the nonrandomized treatment allocation, an exploratory multivariable regression analysis was performed to investigate factors independently associated with healing time, including age, fracture characteristics, and treatment group. Because of the limited sample size and the exploratory retrospective design, advanced adjustment methods such as propensity score matching were not considered statistically reliable. However, exploratory multivariable regression analysis was performed as a sensitivity approach to partially account for potential confounding factors associated with treatment allocation.

A *p*-value < 0.05 was considered statistically significant. Statistical analysis was performed using MedCalc Statistical Software version 14.8.1 (MedCalc Software, Ostend, Belgium).

## 3. Results

### 3.1. Patient Characteristics and Baseline Comparability

A total of 43 patients with aseptic tibial shaft nonunion were included in the study. Fifteen patients were treated with reamed intramedullary nailing alone (Control group), 15 with reamed intramedullary nailing plus teriparatide (Teri group), and 13 with reamed intramedullary nailing plus adipose-derived stem-cell augmentation (ADSC group).

Baseline demographic and fracture-related characteristics were broadly comparable among the three groups without statistical differences. The average age was 41.75 ± 7.24 years in the control group, 41.70 ± 7.35 in the Teri group, and 41.92 ± 7.23 in the ADSC group. The distribution of sex, fracture type, open fracture severity according to Gustilo-Anderson classification, previous fixation method, time from fracture to detection of nonunion, size of the cortical bone defect, ASAMI nonunion subtype, and NUSS score did not differ significantly across groups. (Table 1).

After nonunion surgery, the median follow-up was 2.3 ± 0.64 years in the control group, 2.1 ± 0.62 years in the Teri group, and 2.1 ± 0.62 years in the ADSC group. There was no statistically significant difference between the groups. (Table 2).

### 3.2. Operative Characteristics

The operating time was comparable among the three groups. The mean surgery length was 75.1 ± 33.2 min in the control group, 76.3 ± 28.7 min in the Teri group, and 81.3 ± 29.7 min in the ADSC group. There were no significant differences between groups (*p* > 0.05). Likewise, the mean cortical bone defect size was consistent among groups, indicating a similar level of local bone loss at the nonunion location.

### 3.3. Radiographic Healing

**Figure 1:**
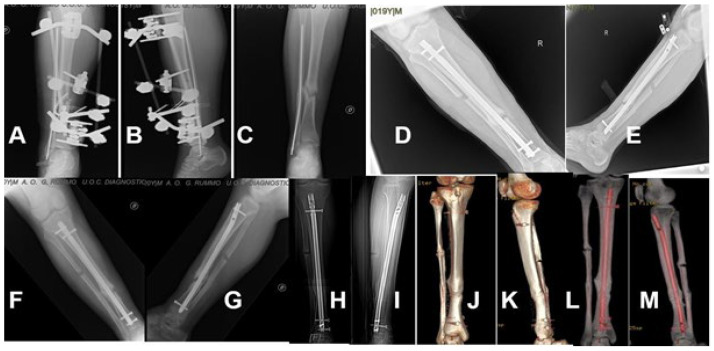
(**A**,**B**) 21-year-old man fallen from motorcycle, open GA IIIB tibia fracture, treated with Modular External Fixator in emergency. (**C**) Aseptic nonunion after 7 months of tibia. (**D**,**E**) Postoperative X-rays, after the reamed nailing and high fibular osteotomy. (**F**,**G**) One month after the surgery the bone callus on 3rd cortex appeared on X-rays. (**H**,**I**) At 3 months, the X-rays showed a good bone callus at the moment of nailing dynamization. (**J**–**M**) At 6 months, 3D CT scan showed the perfect formation of RUST.

**Figure 2:**
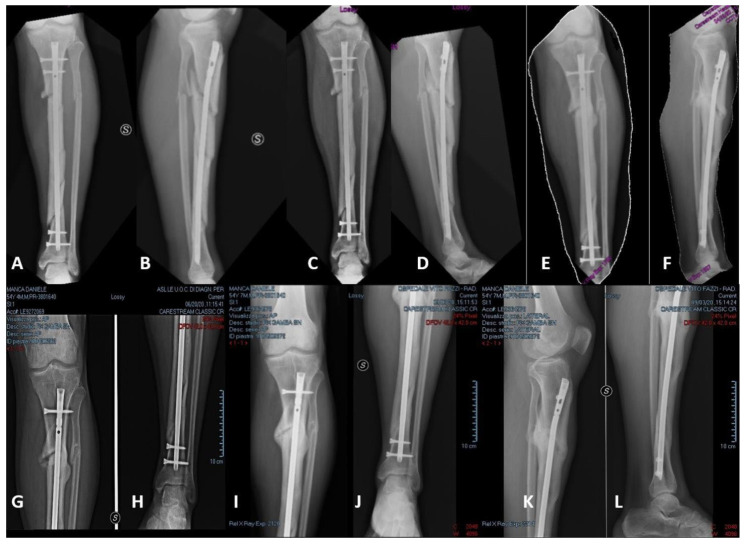
(**A**,**B**) A 49-year-old male after a vehicle accident with multisegmented tibial fractures treated by external fixation and nonunion with reamed nailing. (**C**,**D**) Aseptic nonunion after 3 months of tibia; (**E**,**F**) 1-month X-rays following Teriparatide injection. (**G**,**H**) Three months after the operation, X-rays revealed a bone callus on the third cortex. (**I**,**J**) At four months, X-rays revealed a healthy bone callus at the time of nailing dynamization. (**K**,**L**) At six months, X-rays revealed a hypertrophic proximal bone callus.

**Figure 3:**
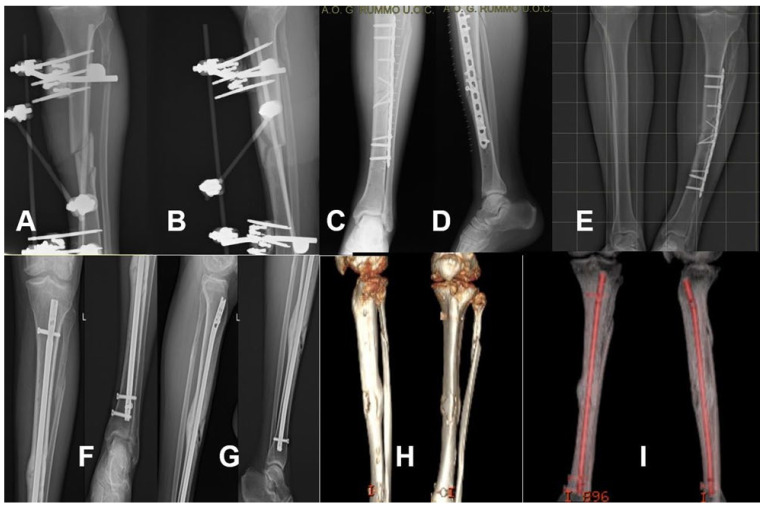
(**A**,**B**) A 43-year-old male crashed off his motorbike, open GA IIB tibia fracture, treated with Modular External Fixator in an emergency. (**C**,**D**) After 21 days of trauma, the patient underwent tibial plating fixation. (**E**) One month after surgery, the plate underwent bending deformation, resulting in a tibial varus. (**F**,**G**) Three months after the nonunion following nailing surgery with ASFC, the X-Rays showed bone callus on the third cortex. (**H**,**I**) At 6 months, the 3D CT scan indicated the perfect creation of RUST.

**Figure 4:**
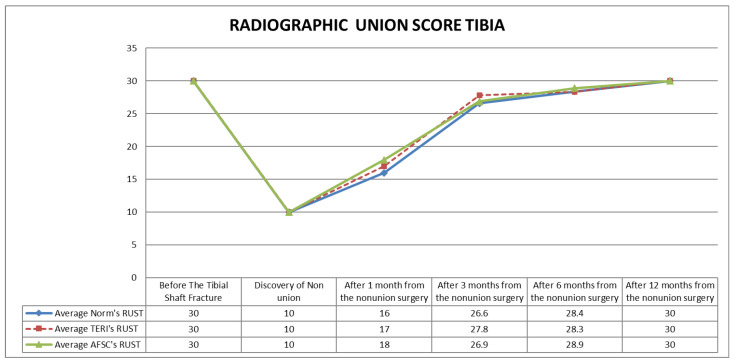
Longitudinal evolution of the Radiographic Union Score for Tibial fractures (RUST) in the Control, Teriparatide (Teri), and ADSC groups during follow-up. Scores were recorded at 1, 3, 6, and 12 months after nonunion surgery. All groups demonstrated progressive radiographic healing over time. No statistically significant between-group differences were observed at any individual follow-up timepoint (*p* > 0.05).

Compared with the control group, teriparatide was associated with a mean reduction in time to tricortical healing of 12.25 days (95% CI: 8.19–16.31), while ADSC augmentation was associated with a mean reduction of 13.30 days (95% CI: 9.52–17.08). The difference between the two augmentation groups was small, with ADSC showing a mean reduction of 1.05 days compared with teriparatide (95% CI: −3.46 to 5.56) as reported in Table 2. Representative cases from the control, teriparatide, and ADSC groups are shown in Figure 1, Figure 2 and Figure 3, respectively. Radiographic healing proceeded over time in all three groups. Serial evaluation using the RUST score showed gradual improvement from the early postoperative period to the final follow-up in each treatment group. No statistically significant between-group differences were observed in RUST values at the scheduled radiographic follow-up assessments. However, all groups showed clear radiographic progression over time, with significant improvement in healing by 12 months compared with earlier follow-up intervals (Figure 4).

Although the overall RUST path was comparable across groups, the period necessary for tricortical radiographic healing differed. The control group had a higher mean time to tricortical repair (99.71 ± 4.29 days) than the Teri group (87.46 ± 6.34 days) and the ADSC group (86.41 ± 5.67 days). This difference was statistically significant (*p* = 0.034), demonstrating that both biological augmentation techniques were associated with quicker radiographic consolidation than nailing alone. (Table 2).

### 3.4. Pain and Its Relationship with Radiographic Healing

**Figure 5:**
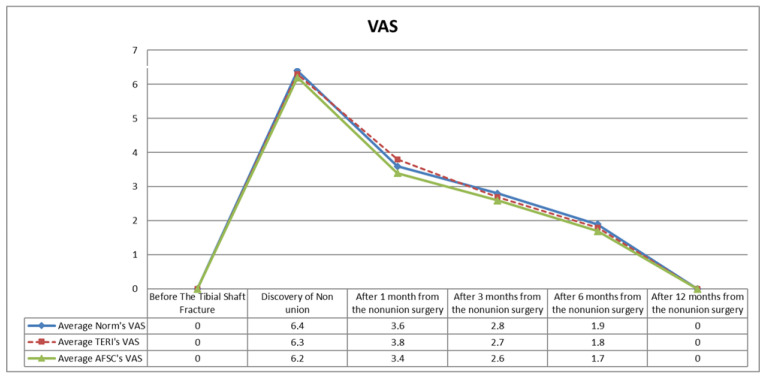
Longitudinal evolution of visual analog scale (VAS) pain scores in the Control, Teriparatide (Teri), and ADSC groups. Pain progressively decreased during follow-up in all groups. No statistically significant between-group differences were observed at any assessment timepoint (*p* > 0.05).

Pain progressively decreased in all three groups during follow-up, as measured by the visual analog scale. The reduction in pain generally paralleled the radiographic progression of healing. Correlation and regression analyses showed a moderate relationship between RUST and VAS values during follow-up, particularly at 3 and 6 months, indicating that better radiographic healing tended to be associated with lower pain levels (Figure 5).

However, when the strength of the RUST–VAS relationship was compared across treatment groups, no statistically significant between-group differences were observed. By the final follow-up, this association was less evident, consistent with the overall clinical improvement observed in all patients.

### 3.5. Complications

The overall complication rate was low and did not differ significantly among groups. Mean intraoperative blood loss was 537 ± 164.38 mL in the control, 587 ± 157.46 mL in the Teri, and 569 ± 155.46 mL in the ADSC group, with no statistically significant difference.

No intraoperative fractures, local skin inflammation, or refractures were recorded during follow-up. Screw loosening occurred in one patient in each group: 1/15 patients (6.66%) in the control group, 1/15 (6.66%) in the Teri group, and 1/13 (7.69%) in the ADSC group. No patient required implant removal during the observed follow-up period. Overall, both adjunctive treatments showed a safety profile comparable to that of nailing alone.

### 3.6. Bone Healing and Functional Outcome

The correlation between radiographic bone regeneration and clinical healing was high in all three groups, with mean concordance values that were comparable across treatment arms (Table 2). Functional outcome at final follow-up, assessed according to the ASAMI classification, was favorable in all groups.

Excellent ASAMI results were recorded in 14/15 patients (93.34%) in the control group, 14/15 (93.34%) in the Teri group, and 12/13 (92.31%) in the ADSC group. The remaining patients in each group achieved a good result. No fair or poor outcomes were observed. There were no statistically significant differences in ASAMI outcome distribution among groups (Table 3).

### 3.7. Quality of Life

**Figure 6:**
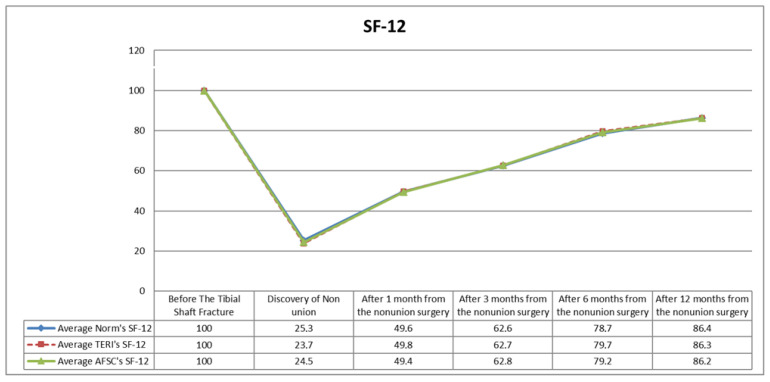
Longitudinal evolution of SF-12 quality-of-life scores in the Control, Teriparatide (Teri), and ADSC groups. Quality-of-life scores improved progressively during follow-up in all groups following treatment of nonunion. No statistically significant between-group differences were observed during the study period (*p* > 0.05).

Health-related quality of life, assessed with the SF-12 questionnaire, followed a similar pattern in all groups. Scores were markedly reduced after the diagnosis of nonunion compared with the pre-injury condition, reflecting the substantial physical and psychological burden of this complication. During follow-up, SF-12 values improved progressively in all three groups. However, no statistically significant differences were found between groups at any follow-up time point (Figure 6).

These findings indicate that, although the addition of teriparatide or ADSC was associated with faster radiographic healing, this earlier consolidation was not accompanied by superior long-term patient-reported quality-of-life outcomes.

### 3.8. Exploratory Adjusted Analysis

An exploratory multivariable analysis was performed to investigate whether selected preoperative variables were associated with healing time. In this model, older age, more complex fracture pattern, and treatment group were associated with time to healing, as shown in Table 4. In particular, the use of biological augmentation showed an association with shorter healing time compared with nailing alone.

Because treatment allocation was nonrandomized and the sample size was limited, this adjusted analysis should be interpreted cautiously and considered hypothesis-generating rather than confirmatory.

No systemic adverse events related to teriparatide treatment, including symptomatic hypercalcemia or treatment discontinuation due to intolerance, were recorded during follow-up. Likewise, no donor-site complications related to adipose tissue harvesting, such as hematoma, infection, persistent pain, or wound healing problems, were observed in the ADSC group. No thromboembolic events, deep surgical site infections, wound dehiscence, or wound-related complications were reported in any treatment group during the study period.

## 4. Discussion

The principal finding of this study was that the addition of teriparatide or adipose-derived stem-cell augmentation to reamed intramedullary nailing was associated with a shorter time to tricortical radiographic healing in patients with aseptic tibial shaft nonunion.

However, long-term functional outcomes, pain improvement at final follow-up, complication rates, and patient-reported quality of life were similar across the three treatment groups. These results suggest that biological augmentation may influence the early phase of radiographic consolidation, but they do not demonstrate a clear long-term clinical advantage over reamed intramedullary nailing alone.

This observation is important from both a scientific and a clinical perspective. In aseptic tibial shaft nonunion, the gold standard of treatment remains correction of instability and restoration of a favorable local environment for healing [6,10,11,12]. Exchange or revision nailing is already known to provide high union rates in properly selected cases, especially when combined with reaming and mechanical optimization [10,11,12]. Our findings should therefore not be interpreted as evidence that teriparatide or ADSCs replace established strategies such as exchange nailing or graft-based biological enhancement in more complex cases. In particular, autologous bone grafting remains one of the most widely accepted biological adjuncts in aseptic nonunion management because of its osteogenic, osteoinductive, and osteoconductive properties. Since the present study did not include a graft-treated comparator group, it is not possible to determine whether the observed acceleration in radiographic healing with teriparatide or ADSC augmentation is comparable, inferior, or superior to standard graft-based strategies [10,11,12].

Rather, this study explores whether two adjunctive biological approaches, applied within the same stable fixation framework, may be associated with earlier radiographic progression in selected patients.

The absence of meaningful between-group differences in final ASAMI or SF-12 outcomes deserves careful interpretation. A faster radiographic healing trajectory does not necessarily translate into better long-term function when all groups ultimately achieve high union rates and favorable clinical recovery. In this setting, mechanical stabilization, early mobilization, and progression to weight-bearing may already provide sufficient conditions for good medium-term recovery, thereby reducing the possibility of finding a later functional separation among groups [30,31]. It is also likely that instruments such as SF-12 and ASAMI, while clinically useful, are not sensitive enough to detect subtle differences in recovery speed once the nonunion has consolidated. Both measures may be affected by ceiling effects in cohorts achieving high union rates and generally favorable outcomes, thereby limiting their ability to discriminate between treatment strategies. Consequently, the absence of significant between-group differences in SF-12 and ASAMI scores should not necessarily be interpreted as evidence of true clinical equivalence.

The biological rationale for the two adjunctive therapies remains plausible, but it should be framed with caution. Teriparatide has been reported to stimulate osteoblast activity, increase callus formation, and support endochondral ossification, and this has led to growing interest in its off-label use for delayed union and nonunion [15,16,17,18,19,32].

A systematic review by Yoon et al. suggested that teriparatide may act as a useful adjuvant in delayed union and nonunion [18], while more recent clinical studies have reported faster healing responses in other fracture settings, supporting its anabolic role in osteoblast activation, endochondral ossification, and callus maturation [19,32]. Similarly, ADSCs have shown regenerative potential through osteogenic differentiation and paracrine signaling, but current human evidence is still based on small and heterogeneous studies, and their clinical role in nonunion treatment remains uncertain [21,23]. Experimental data further suggest that osteogenic regulation may also involve microRNA-mediated pathways, including miR-140-5p, which may influence the differentiation potential of adipose-derived progenitor cells and partially explain their biological activity in impaired bone healing [37].

However, the current clinical literature remains heterogeneous and methodologically limited, particularly in long-bone nonunion. Our findings are therefore better viewed as consistent with these biological hypotheses rather than as proof of efficacy. In other words, the present data support the possibility that these adjuvants may accelerate healing in some patients, but they do not establish that either approach is superior in a definitive therapeutic sense.

Another relevant point is that the present study does not show a difference between teriparatide and ADSC augmentation. Both groups were associated with a shorter mean time to radiographic healing than nailing alone, but their outcomes were otherwise similar. This may indicate that, within the limits of this sample, both interventions act as general biological enhancers rather than as distinct strategies producing clearly different clinical patterns. At the same time, the study is underpowered to identify modest differences between the two adjunctive treatments. Therefore, the absence of a detectable difference between teriparatide and ADSCs should not be interpreted as evidence of equivalence.

The apparent discrepancy between the shorter time to tricortical healing and the absence of significant differences in RUST trajectories at scheduled follow-up timepoints may be partially explained by the interval-based radiographic assessment design. RUST evaluations were performed at predefined postoperative intervals (1, 3, 6, and 12 months), whereas time to tricortical healing was calculated as a time-to-event variable. Consequently, patients achieving radiographic union shortly before or shortly after a scheduled imaging assessment could still present similar RUST scores at fixed follow-up visits despite differences in estimated healing time.

Compared with the current research, our results are directionally matched with publications showing that teriparatide may assist fracture repair and that cell-based treatments may play a role in physiologically deficient bone regeneration [15,18,19,21,23,32]. However, an important limitation of the available evidence, including the present study, is that many reports are based on small cohorts, heterogeneous indications, or non-comparative designs. This makes it difficult to determine whether a positive signal reflects the biological treatment itself, the effect of careful revision surgery, patient selection, or a combination of these factors. Our study adds comparative clinical data in the specific setting of aseptic tibial shaft nonunion treated with reamed intramedullary nailing, but it remains exploratory and should be interpreted within that context.

The study has several important limitations that directly affect the interpretation of comparative treatment efficacy. First, the total sample size was limited for a three-arm comparative study, particularly in the ADSC group (*n* = 13), thereby reducing statistical power and increasing the risk of both type I and type II error. No a priori power calculation was performed because of the retrospective exploratory design. Consequently, the absence of statistically significant differences in secondary outcomes such as ASAMI classification, SF-12 scores, pain, and complication rates should not be interpreted as evidence of equivalence among treatment strategies, as the study may have been underpowered to detect clinically meaningful differences. Regarding the primary outcome, the approximately 12–13 day reduction in time to tricortical radiographic healing observed in the teriparatide and ADSC groups may be clinically relevant, although its real-world impact remains uncertain. Earlier radiographic consolidation could potentially facilitate rehabilitation planning, progression of weight-bearing, earlier return to work, or reduction in the psychosocial burden associated with prolonged nonunion treatment. However, in the present cohort, this radiographic advantage did not translate into superior long-term functional outcomes, quality-of-life scores, or complication profiles. Moreover, no universally accepted minimum clinically important difference (MCID) has been established for time to tricortical healing in aseptic tibial shaft nonunion. Therefore, the observed reduction in healing time should be interpreted as a potentially meaningful signal rather than definitive evidence of clinically superior recovery. Second, treatment allocation was not randomized but depended on clinical contraindications, feasibility of adipose harvesting, and patient acceptance of adjunctive procedures. This introduces a substantial risk of selection bias and residual confounding. Although baseline measured variables appeared comparable among groups, unmeasured factors—including patient motivation, biological healing potential, comorbidity burden, or surgeon-related decision-making—may have influenced both treatment allocation and healing outcomes. Therefore, the observed association between biological augmentation and faster radiographic healing cannot be interpreted as proof of causal superiority over reamed intramedullary nailing alone. For these reasons, the present findings should be considered exploratory and hypothesis-generating rather than confirmatory. Third, the study was retrospective, and some variables, including severity scores, were reconstructed from medical records, which may have introduced classification bias [25]. Fourth, although radiographic healing was assessed systematically, the use of serial imaging still carries an element of observer interpretation despite consensus review [38]. Fifth, the multicenter design increases external relevance but also introduces variability in surgical technique, postoperative management, and, particularly for ADSC use, processing and application methods. This issue is especially important because stromal vascular fraction preparations are not fully standardized across centers, which limits reproducibility [21,23].

An additional methodological limitation relates to the timing of radiographic assessment. Follow-up radiographs were obtained at predefined postoperative intervals (1, 3, 6, and 12 months), rather than through continuous imaging surveillance. Consequently, the exact timing of tricortical radiographic healing may have been influenced by the interval between scheduled assessments. In a relatively small cohort, a patient achieving radiographic union shortly before or shortly after a scheduled imaging visit could influence the calculated mean healing time. Although computed tomography was used in selected cases to support assessment, this interval-based evaluation may still have reduced the precision of healing-time estimation. Another important limitation concerns ADSC characterization. Quantitative information regarding injected volume, cell yield, viability, and final cellular composition was not systematically available because of the retrospective multicenter design. Consequently, the exact biological product administered could not be fully standardized or compared across centers, limiting reproducibility and external validity.

Finally, the study did not include a comparator group treated with alternative biological strategies such as autologous bone grafting, which remains a key reference standard in biologically compromised nonunion. For this reason, the present data do not support conclusions about comparative superiority over established graft-based approaches.

Despite these limitations, the study also has some strengths. It focuses on a clinically relevant and relatively homogeneous problem, namely aseptic tibial shaft nonunion managed with a shared mechanical strategy across all groups. This allows the biological adjuncts to be examined within a consistent fixation framework rather than across fundamentally different reconstructive procedures. In addition, the study reports radiographic, clinical, and patient-reported outcomes over a follow-up period sufficient to assess consolidation and medium-term recovery. From a clinical standpoint, the results may be most relevant for selected patients in whom an earlier radiographic union could facilitate rehabilitation planning, earlier return to work, or reduced psychosocial burden, even if long-term end results remain similar.

Future studies should be designed to address the limitations that remain unresolved here. Larger prospective cohorts or randomized controlled trials are needed to clarify whether the shorter radiographic healing time observed with biological augmentation is reproducible and clinically meaningful. Such studies should use standardized eligibility criteria, clearly defined radiographic endpoints, and more robust adjustment for confounding variables. In the case of ADSCs, standardization of harvesting, processing, cell characterization, and delivery technique will be essential before broader conclusions can be drawn [21,23]. Economic evaluation should also be included, because a treatment that accelerates consolidation but does not improve long-term function may still be useful in selected settings if it reduces indirect costs or recovery time [4,5].

The study should therefore be considered hypothesis-generating rather than definitive, and its main contribution is to support further structured investigation of these biological strategies of biological augmentation within a mechanically optimized treatment strategy.

## 5. Conclusions

Reamed intramedullary nailing remains a reliable mechanical treatment for aseptic tibial shaft nonunion. In the present multicenter retrospective comparative cohort study, the adjunctive use of teriparatide or adipose-derived stem cells was associated with a shorter time to radiographic healing compared with nailing alone. However, this earlier consolidation was not accompanied by superior long-term functional outcomes, complication profiles, or quality-of-life scores.

These findings suggest that biological augmentation may have a role in accelerating radiographic recovery in selected patients, but they do not demonstrate definitive clinical superiority over standard surgical treatment alone or over established graft-based biological strategies. Given the retrospective design, the nonrandomized treatment allocation, the absence of a graft-treated comparator group, and the limited sample size, the results should be interpreted as exploratory and hypothesis-generating.

Further prospective studies with larger cohorts, standardized biological protocols, and direct comparison with autologous bone grafting are needed to better define the real clinical value of teriparatide and adipose-derived stem-cell augmentation in aseptic tibial shaft nonunion.

## Figures and Tables

**Table 1:** Baseline demographic, fracture, and nonunion characteristics of the study groups. Values are reported as mean ± standard deviation or n (%), unless otherwise indicated. *p*-values refer to overall between-group comparisons.

Variable	Control Group (*n* = 15)	Teri Group (*n* = 15)	ADSC Group (*n* = 13)	*p*-Value
Age, years	41.75 ± 7.24	41.70 ± 7.35	41.92 ± 7.23	0.56
Male sex, n (%)	14 (93.3)	14 (93.3)	13 (100)	0.45
Closed fracture, n (%)	5 (33.3)	5 (33.3)	4 (30.8)	0.67
Open fracture, n (%)	10 (66.7)	10 (66.7)	9 (69.2)	0.67
Gustilo I, n (%)	1 (6.7)	1 (6.7)	1 (7.7)	0.75
Gustilo II, n (%)	4 (26.7)	4 (26.7)	3 (23.1)	0.75
Gustilo IIIA, n (%)	5 (33.3)	5 (33.3)	4 (30.8)	0.75
Gustilo IIIB, n (%)	5 (33.3)	5 (33.3)	4 (30.8)	0.75
AO/OTA type A, n (%)	3 (20.0)	3 (20.0)	3 (23.1)	0.56
AO/OTA type B, n (%)	9 (60.0)	9 (60.0)	7 (53.8)	0.56
AO/OTA type C, n (%)	3 (20.0)	3 (20.0)	3 (23.1)	0.56
Previous axial external fixation, n (%)	7 (46.7)	7 (46.7)	5 (38.5)	0.34
Previous circular external fixation, n (%)	2 (13.3)	2 (13.3)	2 (15.4)	0.34
Previous intramedullary nail, n (%)	3 (20.0)	3 (20.0)	3 (23.1)	0.34
Previous plate fixation, n (%)	3 (20.0)	3 (20.0)	3 (23.1)	0.34
Bone defect (one cortex), cm	3.87 ± 0.42	3.82 ± 0.45	3.89 ± 0.47	0.56
ASAMI type B1 nonunion, n (%)	5 (33.3)	5 (33.3)	4 (30.8)	0.85
ASAMI type B2 nonunion, n (%)	6 (40.0)	6 (40.0)	5 (38.5)	0.85
ASAMI type B3 nonunion, n (%)	4 (26.7)	4 (26.7)	4 (30.8)	0.85
NUSS score	52.31 ± 7.24	52.28 ± 7.39	52.31 ± 7.29	0.49

**Table 2:** Perioperative, radiographic, and complication outcomes. Values are reported as mean ± standard deviation or n (%), unless otherwise indicated. The only significant between-group difference was observed for time to tricortical radiographic healing.

Variable	Control Group (*n* = 15)	Teri Group (*n* = 15)	ADSC Group (*n* = 13)	*p*-Value
Follow-up, years	2.3 ± 0.64	2.1 ± 0.62	2.1 ± 0.62	0.57
Operative time, min	75.1 ± 33.2	76.3 ± 28.7	81.3 ± 29.7	0.36
RUST–VAS correlation at 3 months (r^2^)	0.41	0.42	0.43	0.49
RUST–VAS correlation at 6 months (r^2^)	0.77	0.78	0.77	0.67
Time to tricortical radiographic healing, days	99.71 ± 4.29	87.46 ± 6.34	86.41 ± 5.67	0.034
Blood loss, mL	537 ± 164.38	587 ± 157.46	569 ± 155.46	0.67
Screw loosening, n (%)	1 (6.7)	1 (6.7)	1 (7.7)	>0.05
Refracture, n (%)	0	0	0	>0.05
Implant removal during follow-up, n (%)	0	0	0	>0.05
Correlation between radiographic bone healing and clinical healing (κ)	0.97 ± 0.02	0.97 ± 0.03	0.97 ± 0.03	0.58

**Table 3:** Final clinical outcome according to the ASAMI classification. Values are reported as n (%). No significant between-group difference was observed.

ASAMI Outcome	Control Group (*n* = 15)	Teri Group (*n* = 15)	ADSC Group (*n* = 13)	*p*-Value
Excellent	14 (93.3)	14 (93.3)	12 (92.3)	>0.05
Good	1 (6.7)	1 (6.7)	1 (7.7)	>0.05
Fair	0	0	0	>0.05
Poor	0	0	0	>0.05

**Table 4:** Exploratory multivariable regression analysis for predictors of time to tricortical radiographic healing.

Variable	Regression Coefficient (β)	95% CI	*p*-Value
Age	0.31	0.02 to 0.60	0.03
AO/OTA type C fracture	+5.9	1.1 to 10.7	0.02
Bone defect size	+1.7	−0.9 to 4.3	0.18
NUSS score	+0.42	0.09 to 0.74	0.01
Teriparatide group	−10.8	−18.9 to −2.7	0.01
ADSC group	−11.6	−20.1 to −3.2	0.03
Model adjusted R^2^ = 0.41			

## Data Availability

The corresponding author can provide the data described in this study upon request. Due to privacy concerns, the information is not publicly available.
